# Highly Sensitive Humidity Sensors Based on Polyethylene Oxide/CuO/Multi Walled Carbon Nanotubes Composite Nanofibers

**DOI:** 10.3390/ma14041037

**Published:** 2021-02-22

**Authors:** Waqas Ahmad, Bushra Jabbar, Imtiaz Ahmad, Badrul Mohamed Jan, Minas M. Stylianakis, George Kenanakis, Rabia Ikram

**Affiliations:** 1 Institute of Chemical Sciences, University of Peshawar, Khyber Pukhtunkhwa 25120, Pakistan; waqasahmad@uop.edu.pk (W.A.); bushrajabbar93@gmail.com (B.J.); dr_imtiaz@uop.edu.pk (I.A.); 2 Department of Chemical Engineering, University of Malaya, Kuala Lumpur 50603, Malaysia; badrules@um.edu.my; 3 Institute of Electronic Structure and Laser, Foundation for Research and Technology-Hellas, N. Plastira 100, Vasilika Vouton, GR-700 13 Heraklion, Greece; stylianakis@iesl.forth.gr (M.M.S.); gkenanak@iesl.forth.gr (G.K.)

**Keywords:** polyethylene oxide, oxidized multi-walled carbon nanotubes, humidity sensors, copper oxide, composite nanofibers, electrospinning

## Abstract

Polymer composites are favorite materials for sensing applications due to their low cost and easy fabrication. In the current study, composite nanofibers consisting of polyethylene oxide (PEO), oxidized multi-walled carbon nanotubes (MWCNT) and copper oxide (CuO) nanoparticles with 1% and 3% of fillers (i.e., PEO–CuO–MWCNT: 1%, and PEO–CuO–MWCNT: 3%) were successfully developed through electrospinning for humidity sensing applications. The composite nanofibers were characterized by FTIR, XRD, SEM and EDX analysis. Firstly, they were loaded on an interdigitated electrode (IDE), and then the humidity sensing efficiency was investigated through a digital LCR meter (E4980) at different frequencies (100 Hz–1 MHz), as well as the percentage of relative humidity (RH). The results indicated that the composite nanofibers containing 1% and 3% MWCNT, combined with CuO in PEO polymer matrix, showed potent resistive and capacitive response along with high sensitivity to humidity at room temperature in an RH range of 30–90%. More specifically, the PEO–CuO–MWCNT: 1% nanocomposite displayed a resistive rapid response time within 3 s and a long recovery time of 22 s, while the PEO–CuO–MWCNT: 3% one exhibited 20 s and 11 s between the same RH range, respectively.

## 1. Introduction

Recently, tremendous efforts have been made to improve the performance of chemical and physical sensors for technological and daily life applications [1]. In some areas of life, continuous humidity monitoring is essential. Therefore, humidity sensors significantly contribute to various sectors including agriculture, chemical and food production, climate control, environmental monitoring and health, as well as various industrial sectors such as electronics, paper, automobile and pharmaceuticals production [2]. The basic requirements towards the development of excellent and accurate humidity sensors are an exceptionally low hysteresis followed by negligible temperature effect, fast recovery times, thermal stability, long-term durability, resistance to pollutants, low cost and over a wide range sensitivity of RH [3]. A number of materials are used to fabricate humidity sensors such as ceramics, semiconductors and polymers; these sensors follow different mechanisms for measuring the humidity level, which include humidity sensing by change in resistance, capacitance, surface acoustic wave, optical fiber and quartz crystal microbalance [4]. Owing to economical and specific detection mechanisms, capacitive sensors are the most widely used [5]. The mechanism of resistance-dependent humidity sensors hinges on the impedance of the sensing layer, while the capacitive one depends on the dielectric constant.

Moreover, polymer-based humidity sensors offer high sensitivity, small hysteresis, power efficiency, flexibility, low cost and versatile applications. The performance of humidity sensors relies on the sensitivity of sensing materials, which is closely associated with their chemical structure and specific functions. Hence, the sensitivity enhancement of these sensing materials towards humidity is challenging [6]. Very recently, doping of nanomaterials such as metal oxides (i.e., TiO_2_, ZnO, SnO_2_, Al_2_O_3_, CuO) [7,8], carbon-based materials (i.e., carbon nanotubes (CNTs) and graphene) [9] and hydrophilic polymers (i.e., polyethyleneimine (PEI) [10], polyethylene oxide (PEO) [11], polyvinyl alcohol (PVA) [12] and polyaniline (PANI) [13]) have been utilized as composite sensors to obtain an improved sensitivity and response time. 

Over the years, the incorporation of inorganic nanomaterials into polymers has been extensively reported. Metals and metal oxide nanostructures have been paid much attention in the development of sensing devices due to their divergent physical and chemical properties [14]. Metal oxides are preferred over other materials because of their high chemical stability, broad operating temperature range and good mechanical strength [15]. In particular, CuO, which is a p-type semiconducting material, with low bandgap energy (1.2 eV) at ambient temperature, has received much attention. It has been found that CuO can be employed in supercapacitors, field emitters and storage media. Based on its efficient gas sensing and photoelectric properties, CuO is extensively deployed in the fabrication of sensors [16].

On other hand, CNTs have been applied for humidity sensing more widely compared to other carbon-based materials due to their superior electrical, physical and chemical properties. In addition, CNTs possess a large surface-to-volume ratio and nanoscale structure with a hollow core, and hence they are capable of adsorbing large amounts of foreign molecules on the surface [3]. Capacitive and resistive humidity sensors based on oxidized multi-walled carbon nanotubes (MWCNTs) have shown remarkable response and sensitivity, while the addition of CNTs as fillers have improved the electrical and mechanical properties of polymer matrix [9]. In previous studies, ceramics and polymer-based humidity sensors have been tested and compared [17]. Unlike ceramic-based humidity sensors, the polymer-based ones have shown high flexibility, very fast great response time and stability.

A significant variation of permittivity and conductivity among polymer-based humidity sensors can be improved by controlling the porosity and the sensing layer of the polymer matrix [18]. Thanks to their low-cost and easy fabrication, polymers have been extensively used as sensitive materials in different types of sensors [19]. Many studies have demonstrated that polymers can react to any change of humidity up to the micron level of diameter for various industrial applications. Moreover, the response of a polymer-based sensor depends on the analyte’s diffusion into the sensing layer. It was reported that in the case of hydrophilic polymers, the diffusion coefficient of water is low because of the hydrogen bonding and solubility issues [20]; therefore, highly porous polymers with reduced hydrophilicity are being considered more for humidity sensing applications [21]. However, hydrophilic polymers can be used to fabricate high-sensitivity humidity sensors by crosslinking, grafting or by incorporation of a variety of fillers [20,22,23]. Very recently it has been reported that polymeric humidity sensors exhibited unique sensing properties by tuning their electrical characteristics, including the capacitance, at various humidity and conductivity levels as a function of the polymeric moisture content [5]. For instance, the active layer of polyethylene oxide (PEO) is a good candidate for humidity sensing because of the variation in conduction of its ion due to changing the RH [24]. In recent years, due to its toxicity-free nature, water solubility, biodegradability and biocompatibility, PEO has attracted attention for progress in humidity sensing. Therefore, the investigation of a unique sensing material and methods to fabricate high-performance sensors is highly desirable [25]. Compared to conventional methods, the electrospinning technique is a promising and economical method for one-dimensional nanofibers based on metal oxide nanostructures or polymer-based composites [26].

Recently, miniaturized sensors for portable devices such as mobile phones are available, including the following: SHTC1 humidity sensor from Sensirion and LPS331AP pressure sensor from STMicroelectronics are being used in Samsung cell phones; Bosch Sensortec company introduced a miniaturized humidity sensor capable of sensing pressure and temperature (BME280), which are based on Micro-Electro Mechanical Systems (MEMS) [27]. Although the power consumption for these sensors is very small because of their microsize, proportionally, their sensing capacity is also very small, leading to a high signal-to-noise ratio, which requires amplification; likewise, parametric amplification may also be required [28]. All these facts push forward the search for high sensitivity humidity sensors.

The present work demonstrates the realization of low-cost, efficient, flexible and highly stable humidity sensors based on polyethylene oxide, CuO and MWCNTs composite nanofibers by electrospinning. The morphology and structure of composite nanofibers were investigated by SEM, FTIR, EDX and XRD analysis. The humidity sensing performance of the prepared PEO-CuO-MWCNT composite nanofibers was studied through an LCR meter (Inductance, Capacitance and Resistance metre) at fluctuating frequency and RH conditions. The said sensors showed a high capacitive response to humidity, exhibiting high sensitivity, good linearity and fast recovery and response times. Thanks to these remarkable properties, the sensors can be advantageous in several applications such as the monitoring of health and medical facilities, environmental measurements, engineering instruments and remote control of various electronic devices.

## 2. Materials and Methods

### 2.1. Chemicals and Reagents

PEO powder (Merck KGaA; Darmstadt, Germany) with viscosity average molecular weight of 30,000 g/mol. and MWCNTs (diameter and length of 20–30 nm and 10–30 nm, respectively) were provided by National Centre for Physics, Quaid-e-Azam University, Islamabad, Pakistan. Sulfuric acid (H_2_SO_4_) 95% and nitric acid (HNO_3_) 70% were purchased from Merck KGaA; Darmstadt, Germany.

### 2.2. Synthesis of CuO Nanoparticles

CuO nanomaterials were successfully prepared according to a precipitation method [29]. A stoichiometric amount of copper sulfate pentahydrate (CuSO_4_^.^5H_2_O; Merck KGaA; Darmstadt, Germany) was dissolved in a calculated volume of distilled water to prepare a solution (0.2 M). The CuSO_4_ solution was taken in a beaker and placed on a magnetic stirring hot plate, with a pH electrode inserted in the solution. Then, a solution of NaOH (2.0 M) was added dropwise into the CuSO_4_ solution under continuous stirring. The addition of NaOH was continuously done until a pH of 8.5 was obtained. The solution was further stirred at 90 °C for 1 h, and then the resultant precipitate was recovered by filtration followed by washing with distilled water. The precipitate was calcined in a muffle furnace at 400 °C for 4 h. Finally, a dark brown powder of CuO was obtained, which was cooled in a desiccator and stored in vials.

### 2.3. Oxidation of MWCNTs

MWCNTs were oxidized by an acid treatment using a procedure reported elsewhere [30]. MWCNTs were dispersed in a mixture of concentrated H_2_SO_4_ and HNO_3_ (3:1) and sonicated in an ultrasonic bath for 3 h at 40 °C. The suspension was recovered by means of filtration and washed with distilled water until the pH to be neutral. The MWCNTs were then dried under vacuum in an oven at 50 °C for 5 h. 

### 2.4. Preparation of the Polymer Composite Blend

The PEO-CuO-MWCNT composite blend was prepared by incorporating CuO nanoparticles and oxidized MWCNTs as fillers in PEO polymer matrix. To prepare blend solution, PEO was initially dissolved in distilled water and stirred for 1 h, and then the calculated weights of CuO nanoparticles and MWCNTs required for a ratio of 1:1 and 1:3 (i.e., in PEO-CuO-MWCNT:1% and PEO-CuO-MWCNT:3% nanocomposites) were added to the PEO solution, respectively. The mixture was homogenized by ultrasonication for approximately 4 h at 40 °C. Finally, the resulting homogenous dispersion with the dark brown appearance was continuously stirred to get a viscoelastic solution for the synthesis of nanofibers [31].

### 2.5. Nanofibers Development via Electrospinning

The composite nanofibers were synthesized by electrospinning. The PEO–CuO–MWCNT solution was loaded in a 10 mL syringe fixed with a 21 gauge blunt needle linked to a high-voltage power supply. The positive electrode of the power supply was connected to the needle, while the negative one was connected to an interdigitated electrode (IDE). Aluminum foil was also attached to the negative electrode in order to collect the nanofiber for further characterization. The distance between the positive electrode (syringe) and the collector was 15 cm, and high electrostatic voltage was applied to synthesize the nanofibers at a flow rate of 0.5 mL/h by a syringe pump leading to the formation of a droplet of the composite solution. At a voltage of 15 Kv, the solution was sprayed on the Al foil and IDE screen, composite nanofibers were formed as the solvent evaporated. Graphical presentation is given in Figure S1 (Supplementary Materials).

### 2.6. Characterization of PEO–CuO–MWCNT Composite Nanofibers

XRD patterns of pure PEO, CuO nanoparticles, MWCNTs and PEO–CuO–MWCNT nanofibers were performed using an X-ray diffractometer (Model JDX-9C, JEOL; Akishima, Tokyo, Japan) at room temperature. The morphology of the samples was examined by scanning electron microscope (Model JEOL-JSM-5910; Akishima, Tokyo, Japan), while the elemental analysis of the composite and the precursor materials was investigated by EDS analysis (Oxford Instruments, High Wycombe, United Kingdom). Fourier transform infrared (FTIR) spectra of pure PEO, CuO nanoparticles, MWCNTs and nanocomposites were taken through an FTIR spectrophotometer (Shimadzu FTIR-820.1 PC; Tokyo, Japan). 

### 2.7. Humidity Sensing Experiments

The humidity sensing properties of the composite nanofibers were studied by measuring their capacitance and resistance at different RH levels using a digital LCR meter (Keysight E4980; Keysight Technologies, Santa Clara, CA, USA). The reference humidity sensor (DHT11) was combined with a microcontroller (ARDUINO UNO 328; Somerville, MA, USA), and both were connected to a computer through an RS232 card. The composite nanofibers were loaded on the interdigitated electrode (IDE) and connected to the LCR meter. The IDE electrode was placed in a moisture chamber along with the reference sensor, while atmospheres containing various humidity levels were adjusted passing dry nitrogen and air saturated with vapors of deionized water. The moisture chamber was sealed, and the change in resistance and capacitance was recorded at every 60 s under varying RH between 30 and 90%. The values of the resistance and capacitance changes with % RH were recorded and digitized, respectively.

## 3. Results and Discussion

### 3.1. Characterization of Composite Nanofibers

#### 3.1.1. SEM Analysis

**Figure 1:**
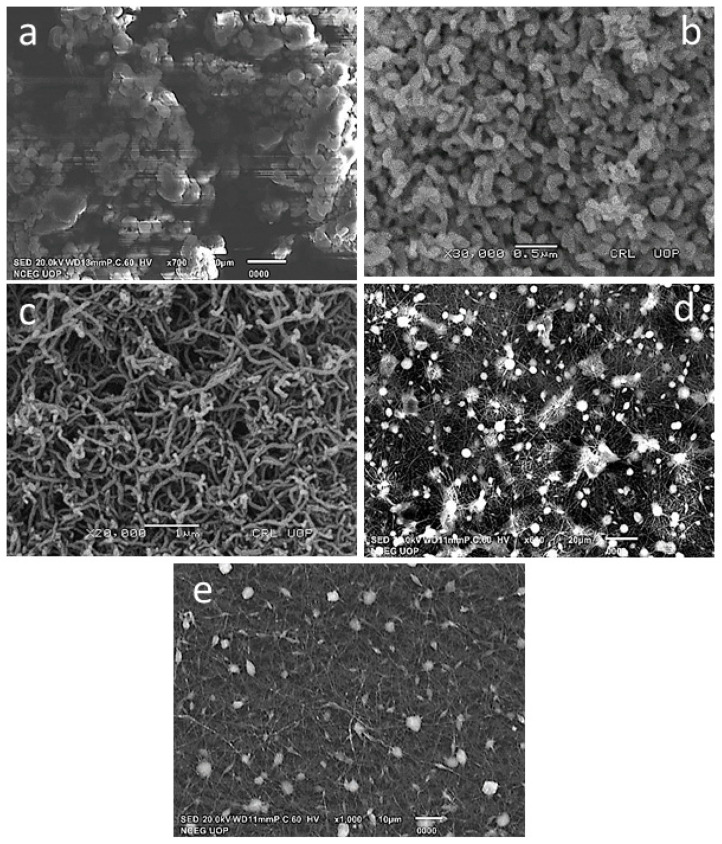
SEM micrographs of (**a**) pure PEO (**b**) CuO nanoparticles (**c**) multi-walled carbon nanotubes (MWCNT) (**d**) PEO: CuO: MWCNT: 1% nanofibers (**e**) PEO: CuO: MWCNT: 3% nanofibers.

The morphological features of the composite nanofibers were examined by Scanning Electron Microscopy (SEM). Figure 1a–d shows the SEM micrographs of pure PEO, CuO nanoparticles, MWCNTs and PEO–CuO–MWCNT nanocomposites. The SEM image of pure PEO is displayed in Figure 1a, revealing a compact flaky morphology; the granules seem to be agglomerated and highly dense, thus confirming its crystalline nature as also indicated by XRD analysis [32]. Figure 1b displays the micrograph of CuO nanoparticles, which shows a homogeneous distribution of fine spherical particles of CuO nanoparticles [33]. The micrograph of MWCNTs (Figure 1c) shows that this highly intertwisted configuration could be assigned to the functionalization of MWCNTs [34]. The SEM micrographs of PEO–CuO–MWCNT: 1% and PEO–CuO–MWCNT: 3% (Figure 1d,e) exhibit very fine fibers with uniform and smooth surfaces, indicating the uniform dispersion fillers in the polymer matrix. However, in the case of PEO–CuO–MWCNT (3%), some agglomerates may be observed, which may be caused due to high amounts of CuO and MWCNT. 

#### 3.1.2. X-ray Diffraction Analysis

The X-ray diffraction (XRD) patterns of pure PEO, CuO nanoparticles, MWCNTs and composite nanofibers are illustrated in Figure S2 (Supplementary Materials). The XRD pattern of pure PEO (Figure S2a) shows two sharp peaks at 19.22° and 23.1°, attributed to its highly crystalline structure [35]. The XRD pattern of CuO nanoparticles (Figure S2b) confirmed the two characteristics peaks at 35.57° and 38.85°, showing a high crystallinity phase of CuO [36]. Finally, the XRD pattern of MWCNTs (Figure S2c) shows a sharp and strong diffraction peak at 25.70° [37], attributed to the crystalline phase of CNTs.

In the XRD patterns of PEO–CuO–MWCNT: 1% and PEO–CuO–MWCNT: 3% (Figure S2d,e), the characteristic peaks corresponding to PEO and MWCNT can be observed. In the case of PEO–CuO–MWCNT: 1%, the crystalline peaks for MWCNT and CuO are less evident due to the lower concentration ratio of the fillers in the PEO matrix. However, the XRD pattern of PEO–CuO–MWCNT: 3% retains the crystalline peaks of CuO and MWCNT. Hence, the crystallinity of the composites seems to be maintained after the addition of MWCNTs and CuO nanoparticles [38].

#### 3.1.3. FTIR Spectroscopy

**Figure 2:**
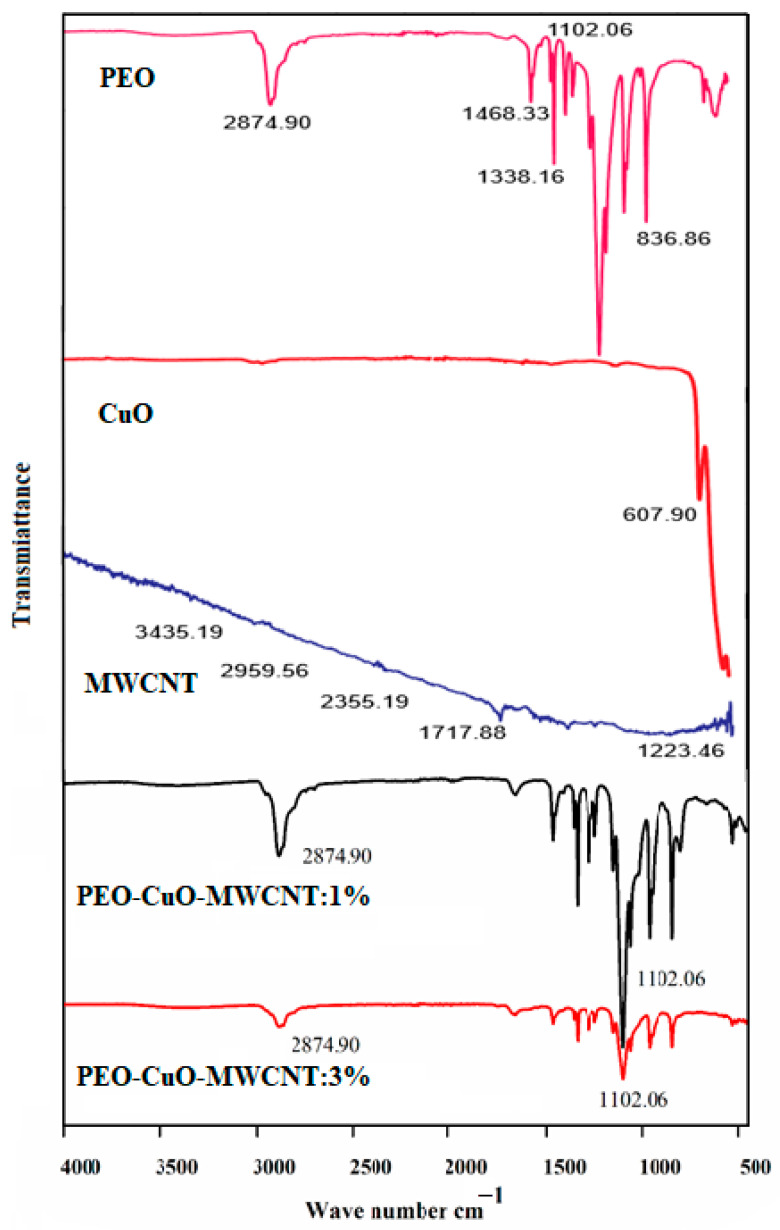
FTIR spectra of pure PEO (purple), CuO nanoparticles (red) and MWCNTs (blue), PEO–CuO–MWCNT: 1% (black) and PEO–CuO–MWCNT: 3% (orange) composite nanofibers.

The FTIR spectra of the polymer matrix, i.e., PEO, fillers (CuO and MWCNTs) and the composite nanofibers, are shown in Figure 2. The FTIR spectrum of pure PEO shows a strong peak at 2874.90 cm^−1^, which is related to asymmetric C-H stretching vibrations of CH_2_ groups. The significant central peak at 1468.33 cm^−1^ is due to asymmetric bending vibrations of CH_2_ groups, while a strong intense peak at 1102.06 cm^−1^ is attributed to the asymmetric C–O–C stretching for epoxide groups. These configurations are in agreement with the structural features of PEO polymer [39]. The FTIR spectrum of CuO nanoparticles is shown in (Figure 2), presenting a major peak at 607.90 cm^−1^ [40]. The spectrum of MWCNTs shows two strong bands at 3435 and 1717 cm^−1^, representing the O–H and C=O of carboxylic acids, respectively [37], this indicates the successful oxidation of MWCNTs.

The spectra of PEO–CuO–MWCNT: 1% and PEO–CuO–MWCNT: 3% nanocomposites are displayed in Figure 2. It is obvious that FTIR spectra for both composites show peaks corresponding to CuO, MWCNT and PEO, confirming the presence of these components in the composite samples. However, it should be noted that, in the case of PEO–CuO–MWCNT: 3% nanocomposite, the intensity of the peaks positioned at 2874.90 and 1102.06 cm^−1^ is slightly decreased, which may be attributed to the interaction between the ether group of the poly ethylene oxide and the hydroxyl one of the oxidized MWCNT, which are linked through hydrogen bonding [41]. Since the amount of MWCNT is higher in the composite containing filler 3%, the extent of interaction is larger, causing an obvious decrease in peak intensity compared to the nanocomposite with 1% MWCNT. All the configurations in the nanocomposite samples give peaks at respective wavelengths, as mentioned above [41].

#### 3.1.4. EDS Analysis

Figure S3a–d displays the energy dispersive spectroscopy (EDS) profiles and the atomic ratio of MWCNTs, pure polyethylene oxide (PEO) and composite nanofibers PEO–CuO–MWCNT. The elemental composition of MWCNTs confirms the presence of about 84% of C and 16% of O. The significant amount of oxygen and carbon suggests the successful oxidation by nitric acid and sulfuric acid, along with some impurities of Al and Si as residues of the various reagents involved during the chemical treatment. EDS analysis of pure PEO matrix shows about 58.50% carbon and 36% carbon, confirming the composition of PEO [42]. The elemental composition of composite nanofibers, i.e., PEO–CuO–MWCNT: 1% and PEO–CuO–MWCNT: 3% shows the presence of C, O and Cu, which is in agreement with their chemical composition [43]. In the case of PEO–CuO–MWCNT: 1% nanofiber, the amount of Cu is shown to be 0.5%, whereas in that of PEO–CuO–MWCNT: 3%, it is ~1.89%. Although the values seem to be slightly lower than the theoretical values of 1 and 3%, this behavior might be attributed to the low sensitivity of the EDS analysis in a very low concentration of elements.

### 3.2. Humidity Sensing Efficiency of PEO–CuO–MWCNT Composite Nanofibers

The humidity sensing performance of PEO–CuO–MWCNT composite nanofibers was investigated by measuring its capacitance and resistivity response in various levels of RH as a function of frequency. Initial experiments showed that PEO– composite nanofibers with 1% CuO presented a significant resistivity response, better than the respective capacitance response upon changing the RH. 

#### 3.2.1. Sensitivity and Response

**Figure 3:**
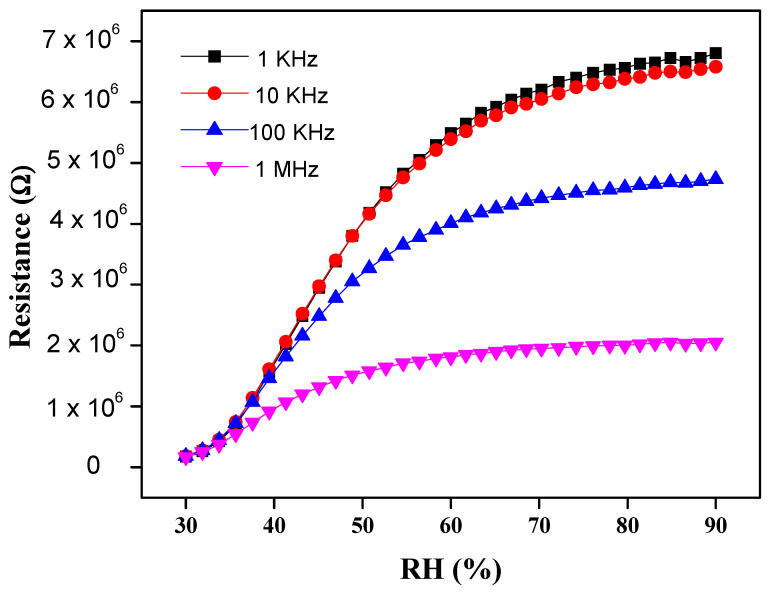
Resistivity response of PEO–CuO–MWCNT: 1% nanocomposite at different % RH.

**Figure 4:**
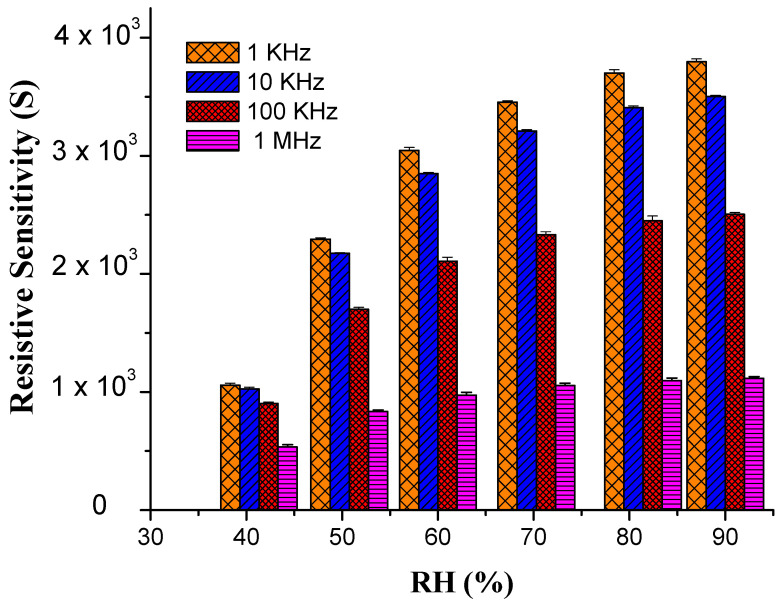
Resistivity Sensitivity of PEO-CuO-MWCNT:1% nanocomposite at different % RH.

**Figure 5:**
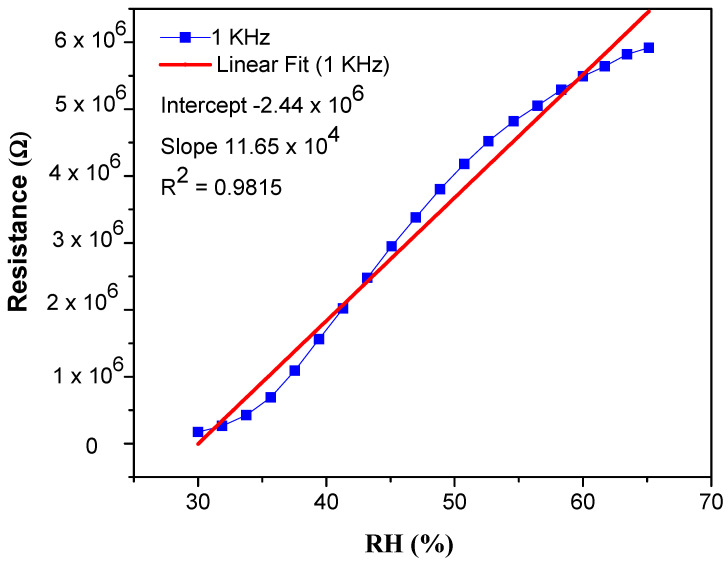
Resistive linearity of PEO−CuO−MWCNT: 1% nanocomposites.

**Figure 6:**
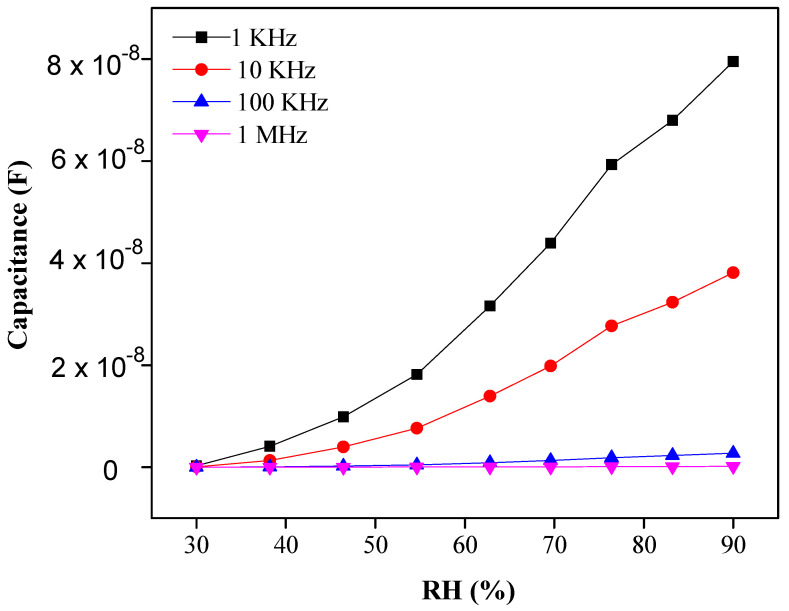
Capacitance response of PEO−CuO−MWCNT: 3% nanocomposite at different % RH.

**Figure 7:**
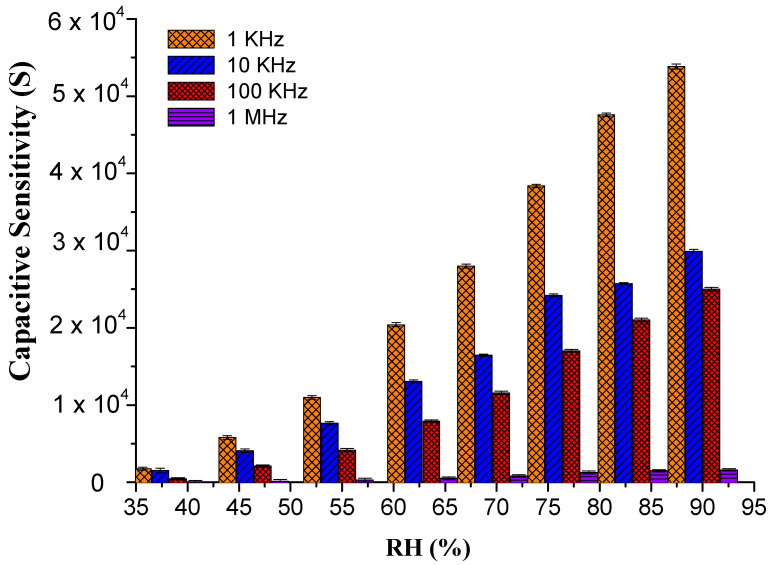
Capacitive Sensitivity of PEO−CuO−MWCNT: 3% nanocomposite at different % RH.

**Figure 8:**
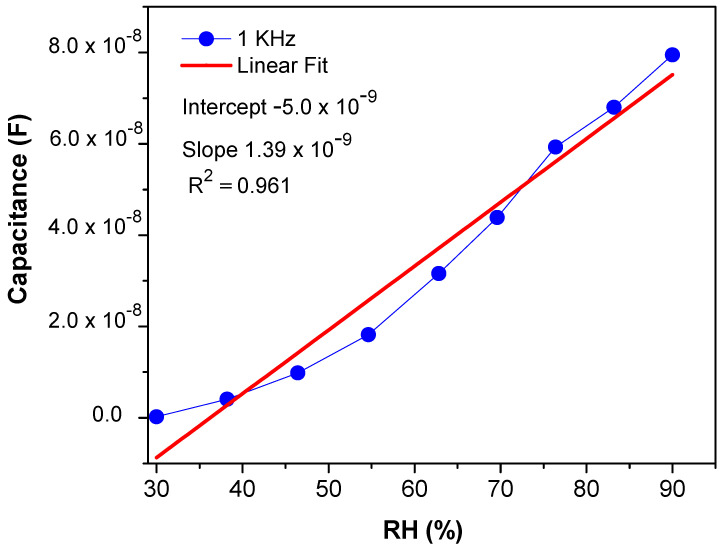
Capacitive linearity of PEO−MWCNT−CuO: 3% nanocomposites.

It has been shown that frequency has a major role and impact on the humidity sensing performance. The results of humidity sensing efficiency of the composite nanofibers are investigated at various input frequencies such as 1 KHz, 10 kHz, 100 KHz and 1 MHz. Figure 3, Figure 4, Figure 5, Figure 6, Figure 7 and Figure 8 indicate the resistivity and capacitance response, of PEO–CuO–MWCNT: 1% and PEO–CuO–MWCNT: 3% composite nanofibers upon % RH, ranging from 30–90%. When RH increases, the capacitance and resistance of nanocomposites also increase along with the frequency. Conversely, it is observed that at low frequencies, the sensor shows high capacitive and resistive response, as compared to high frequency [44]. The following equation is used to determine the sensitivity of the sensor:

(1) $$\mathrm{Resistive}\;\mathrm{Sensitivity}\;\left(S\right)=\frac{\Delta R/R_{0}}{\Delta \left(\%\mathrm{RH}\right)}$$

(2) $$\mathrm{Capacitive}\;\mathrm{Sensitivity}\;\left(S\right)=\frac{\Delta C/C_{0}}{\Delta \left(\%\mathrm{RH}\right)}$$

where ∆R and ∆C are the changes in resistance and capacitance when exposed to fluctuating humidity values with respect to initial value R_0_ or C_0_.

Our results showed that the resistance and capacitance of the sensor significantly increased when RH value changed from 30–90% RH. This significant increase in capacitance could be associated with the adsorption of water molecules on the surface of the sensor, which directly depends on the RH level [4]. In the adsorption process, due to an electrical potential gap, part of the electrons will be moved from the adsorbed H_2_O molecules to MWCNTs. The higher the amount of RH, the greater the adsorption of H_2_O molecules and the more electrons are transferred. The PEO–MWCNT–CuO behaves as a semiconductor, containing electrons (−vie charges) and holes (+ive charges). In the present case, the electrons are minority carriers and the holes are the majority carrier. As electron (minority carrier) from water molecules are transferred, this will lower the concentration of majority carrier, i.e., +ive charges (holes), thereby resulting in an increase in the resistance of the sensor [45]. 

The remarkable increase of resistivity response was measured over a % RH range from 30 to 90% at four different frequencies, i.e., 1 KHz, 10 KHz, 100 KHz and 1 MHz, respectively. It was observed that the resistance at lower frequencies (1 KHz and 10 KHz) enhances proportionally to RH. This resistance change of the sensor is displayed in Figure 3. The data show that at 30% RH, the resistivity shown by 1% wt nanocomposite is about 1.5 × 10^5^ Ω, which is smaller than 6.7 × 10^6^ Ω at 90% RH at 1 KHz frequency [46]. At low frequencies, the PEO–MWCNT–CuO: 1%-based sensor exhibited the highest resistivity sensitivity of ~3798.2%, as displayed in Figure 4, with a slightly low correlation in resistance (R^2^) as compared to PEO–MWCNT–CuO: 3% nanocomposite-based sensor, as shown in Figure 5.

The capacitance response of PEO–CuO–MWCNT: 3% nanocomposites drastically increased from 1.09 × 10^−10^ F (for 30% RH) to 7.9 × 10^−8^ F (for 90% RH) as shown in Figure 6, while a frequency shift can be observed from 1 KHz to 1 MHz [47]. The high capacitance response of PEO–CuO–MWCNT: 3% composite nanofibers can be ascribed to the formation of multilayers of water on the exterior of the sensor that increases the dielectric constant, leading to an abrupt capacitance change [48]. However, the ideal situation for a sensing material is revealed when the humidity is low, and thus less water is absorbed. Subsequently, leak conduction occurs by absorbing water molecules. The capacitance C of the sensing material with leak conduction can be illustrated by the following equation:

(3) $$C\;=E\;*\;C_{0}\;=\;ℰr–\;ί\;\gamma \;/\omega E_{0}\;C_{0}$$

where ℰ is the complex dielectric constant, C_0_ and Er are capacitance and relative dielectric parameter of an ideal capacitor, Ꞷ stands for frequency, γ is the conductivity and ℰ_0_ presents the permittivity in free space.

From Equation (3), it can be concluded that the capacitance of the sensing material is directly proportional to γ and inversely proportional to frequency [49]. Moreover, Figure 7 shows the sensor exhibited high capacitive sensitivity of ~53,837.6% at lower frequencies such as 1 KHz at 90% RH [50] than ~3784.81% resistivity sensitivity. Though the sensor shows a high sensitivity within the RH range of 30–70%, the response or sensitivity in the RH range of 70–90% RH is also considerable. PEO–CuO–MWCN: 3% nanocomposite showed improved linear regression coefficient (R^2^) with a positive slop over wide humidity range levels such as 30–90% RH as displayed in Figure 8.

#### 3.2.2. Response and Recovery Time

**Figure 9:**
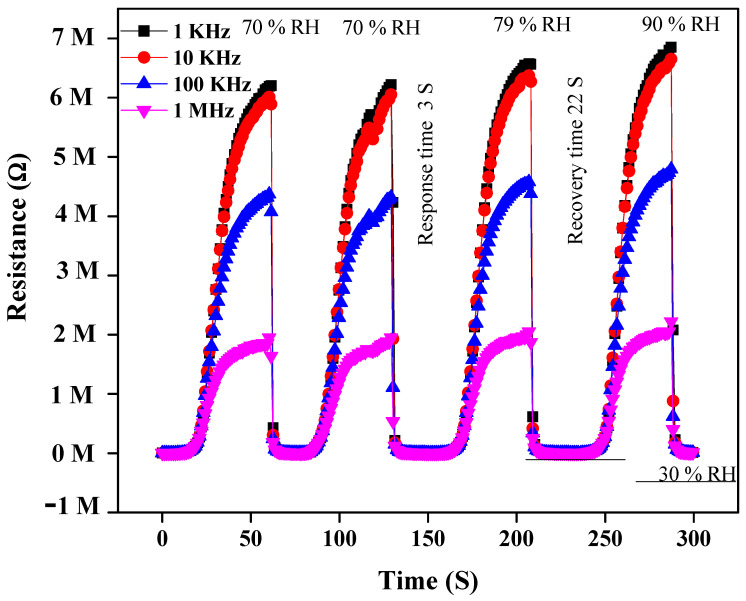
Resistivity response/recovery time for PEO−CuO−MWCNT: 1% nanocomposites.

**Figure 10:**
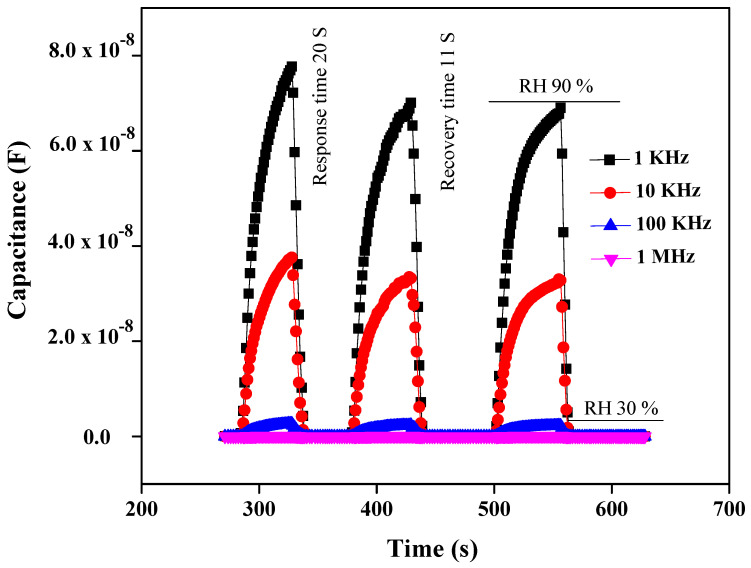
Capacitance response/recovery time for PEO−MWCNT−CuO: 3% nanocomposites.

One of the considerable parameters for the fabrication of excellent and economical humidity sensors is their fast response and recovery time. Usually, response and recovery times are measured in 10–90% RH, but in the present case, the response of the sensor below 30% RH was negligible; hence, measurement was carried out between 30 and 90% RH. When air humidity is suddenly varied from 30 to 90% RH, the response and recovery time can be verified by the capacitive and resistive variation of the humidity sensor. The response or recovery time is the time responsible for causing a 90% change of their original value when increasing the humidity from a low to a high degree or vice versa, respectively. Due to the hydrophilic nature of the PEO matric, and its large surface area, it is perceived that composite nanofibers allow faster adsorption of water molecules. In this context, the response and recovery time of resistivity and capacitance response of PEO−CuO−MWCNT: 1% and PEO−CuO−MWCNT: 3% composite nanofibers have been presented in Figure 9 and Figure 10, at four different frequencies, from 30% to 90% RH. The response time for resistivity response is measured to be 3 s and 22 s respectively when humidity changes from 30 to 90%. The response and recovery time for a capacitance humidity sensor is measured to be 20 s and 11 s respectively, for 30 to 90% RH. 

Owing to their low density and high aspect ratio, carbon nanotubes have proven to be ideal fillers for designing diverse polymer composites with enhanced mechanical performance, high electrical conductance and multi-functional properties [51]. Additionally, the oxyfunctionalities incorporated during the oxidation of MWCNTs may provide more active sites for the interaction of water molecules. Likewise, CuO is assumed to enhance the interfacial characteristics and porosity of the polymeric matrix, enabling it to efficiently absorb the water molecules [52]. Since PEO is a hydrophilic polymer, the incorporation of CuO nanoparticles and MWCNTs into it results in nanocomposites that exhibit a fast response. CuO nanoparticles and MWCNTs provide large surface-active sites for the adsorption of moisture [53]. This adsorption occurs due to the functional groups of MWCNTs and PEO, through hydrogen bonds formation or through weak physical forces with the adsorption site in the large surface area of nanocomposites [54]. However, the hydrophilic nature of the composites delays the water release; therefore, recovery time was longer at 22 s and 11 s. At high frequencies (1 MHz or 100 KHz), the response and recovery time is much lower for the capacitance humidity sensor, than the resistivity humidity sensor, as depicted in Figure 9 and Figure 10. 

#### 3.2.3. Humidity Sensing Mechanism

**Figure 11:**
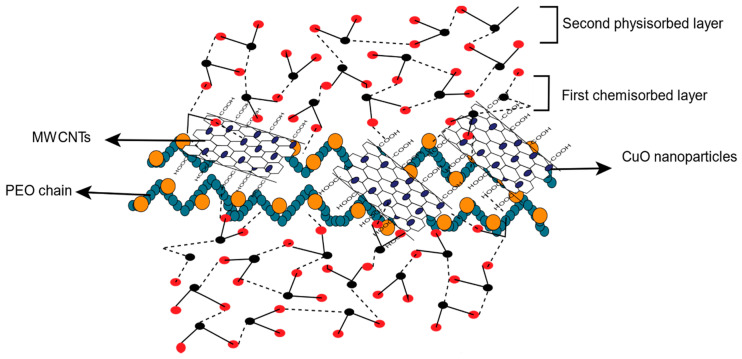
Schematic presentation of adsorption of H_2_O molecules on the surface of PEO−MWCNT−CuO nanocomposites.

The humidity sensing mechanism for the adsorption of water molecules on the surface of PEO−CuO−MWCNTs is displayed in Figure 11. The rough and smooth surface of nanocomposites can be indirectly detected by the addition of CuO nanoparticles inside the hydrophilic polymer matrix to improve the large surface area of the nanocomposite, which enables more water molecules to be adsorbed on the sensing material [5]. On the contrary, the formation of hydrogen bonds between hydrophilic PEO and water molecules enhances the humidity sensing response [10]. Moreover, the large surface area of MWCNT nanostructures as well as the incorporation of −COOH functional side groups create active sites for the adsorption of vapor molecules [55].

To explain the humidity sensing mechanism, two types of adsorption interactions were considered i.e., chemisorption and physisorption [4]. The current study initiated at low RH the nanofiber composite exposed to water molecules. The additional water molecules were adsorbed on the surface of the hydrophilic PEO through intermolecular hydrogen bonding, identified as chemisorbed layer [48]. With increasing RH level, a water multilayer was formed between two adjacent hydroxyl groups via hydrogen bonding, leading to the formation of a physisorption layer [56]. Water molecule clustering was also noted [57]. However, since the increase in capacitance is very small as the RH increased from 30 to 50%, it was concluded that water molecules adsorbed in the first layer had a very small contribution to the dielectric constant. This can be attributed to the fact that chemisorbed water molecules cannot be freely oriented with an external applied electric field, due to their bonding (hydrogen bonding) with composite nanofibers [58], which leads to very small change in dielectric constant and thereby causes little variation in capacitance. Therefore, it was observed that there is significant increase in capacitance around 50% RH. This indicates that at 50% RH, the first layer was almost completed and the second layer’s formation has begun. Hence, the capacitance and resistance are drastically increased between 50 and 90% RH due to the formation of a multilayer in the sensing material. Since the PEO−MWCNT−CuO: 1% composite nanofibers exhibited resistive response whereas PEO−MWCNT−CuO: 3% showed a capacitive response, this indicates that with the increasing of the filler’s concentration, the behavior of the composite changed from resistive to capacitive. This may be attributed to the conductive nature of both fillers, i.e., MWCNT and CuO, in the case of PEO−MWCNT−CuO: 1% nanocomposites where the concentration of CuO was smaller, and hence the conductance is less, and the dielectric constant was negligible. Consequently, due to very low dielectric constant, the resistivity of the composite is higher than its capacitance, which is why it shows high resistive response. On the other hand, in the case of the PEO−MWCNT−CuO: 3% nanofibers-based sensor, the high concentration of conducting fillers leads to increased dielectric constant, due to which the sensor exhibits capacitive response with varying RH. 

### 3.3. Comparative Efficiencies of the Sensors 

Table 1 displays the comparison between the resistive and capacitive response to % RH different sensor materials reported in the literature, along with their recovery and response time. It is evident that the sensor developed in the current study i.e., PEO−MWCNT−CuO: 1% and PEO−MWCNT−CuO: 3%, exhibited much higher sensitivity of 3798.2% and 53837.6% with response and recovery times of 3 s and 22 s and at 20 s and 11 s, respectively, for 30% to 90% RH. Thus, the comparative analysis concludes that the sensors used in this study showed higher sensitivity in relation to other previously reported humidity sensors. Finally, the response and recovery time of current sensors are rapid compared to the reported ones in the literature. Thus, these humidity sensors can be potentially used for practical applications. 

## 4. Conclusions

A novel highly responsive humidity sensor was fabricated based on PEO−CuO−MWCNTs composite nanofibers. The composites nanofibers were developed for capacitive and resistive humidity applications prepared via electrospinning. The composite nanofibers PEO−CuO−MWCNT: 1% and PEO−CuO−MWCNT: 3% humidity sensor revealed high sensitivity with fast response and recovery time. We found that the PEO−CuO−MWCNT: 1% composite nanofibers showed high humidity sensitivity (3798.2%) and exhibited a quick response of 3 s and recovery time of 22 s. It was observed that in PEO−CuO−MWCNT: 1% composite nanofibers, the resistance increased linearly when exposed to humidity. The PEO−CuO−MWCNT: 3% nanocomposite showed a response of 20s and a rapid recovery time of 11s with high sensitivity (53837,6%), while the capacitance increased as the frequency decreased. All results demonstrate that increasing the concentration of fillers i.e., MWCNTs and CuO nanoparticles is an effective approach to improve the sensing properties. The proposed method allows the scalable production of multicomponent sensor devices based on polymer matrix composite with high stability, flexibility and versatility for several industrial applications.

## Figures and Tables

**Table 1:** A comparison of the resistive and capacitive response to RH.

Sensing Material	Type	RH (%)	Sensitivity	R^2^	Response/Recovery Time	Ref.
GO/MWCNT	Capacitive	11–97	7980%	-	5 s /2.5 s	[59]
MWCNT/Nafion nanofibers film	SAWR	10–80	427.6%	0.987	3 s at 63%	[60]
PANI/PVB nanofibers	SAWR	20–90	∼75 kHz/%RH	0.927	1/2	[19]
s2DMoS^2^	Resistive	0–80	85 KΩ%/RH	0.999	0.6/0.3	[61]
Poly-AMPS/ TEOS	Resistive	30–90	-	0.998	<2 min	[62]
Organic silicon sol/poly-AMPS	Resistive	30–90	-	0.9491	30/60 s	[63]
PEO−CuO−MWCNT: 1%	Resistive	30–90	3798.2%	0.884	3/22 s	Current Study
PEO−CuO−MWCNT: 3%	Capacitive	30–90	53837.6%	0.961	20/11 s	Current Study

## Data Availability

All data reported here can be made available on request.

## Supplementary Materials

The following are available online at https://www.mdpi.com/1996-1944/14/4/1037/s1, Figure S1: The schematic diagram of the Electrospinning process; Figure S2: The XRD patterns of (**a**) pure PEO (**b**) CuO nanoparticles (**c**) MWCNT (**d**) PEO–CuO–MWCNT: 1% nanocomposite and (**e**) PEO–CuO–MWCNT: 3% nanocomposite; Figure S3: EDS elemental profiles of (**a**) MWCNT (**b**) pure PEO (**c**) PEO–MWCNT–CuO: 1% nanofibers, and (**d**) PEO–MWCNT–CuO: 3% composite nanofibers.
