# Supplementary material for: Highly Sensitive Humidity Sensors Based on Polyethylene Oxide/CuO/Multi Walled Carbon Nanotubes Composite Nanofibers
Source: Materials (Basel). 2021 Feb 22;14(4):1037. doi: 10.3390/ma14041037 (PMC7927036; doi:10.3390/ma14041037)
Supplement: Supplementary file 1 [file materials-14-01037-s001.zip › materials-1097603-supplementary.pdf]

materials

MDPI

Article

Highly Sensitive Humidity Sensors Based on Polyethylene Oxide/CuO/Multi Walled Carbon Nanotubes Composite Nanofibers

Waqas Ahmad [1], Bushra Jabbar [1], Imtiaz Ahmad [1], Badrul Mohamed Jan [2], Minas M. Stylianakis [3], George Kenanakis [3] and Rabia Ikram [2,*]

[1] Institute of Chemical Sciences, University of Peshawar, 25120 Khyber Pukhtunkhwa, Pakistan; waqasahmad@uop.edu.pk (W.A.); bushrajabbar93@gmail.com (B.J.); dr_imtiaz@uop.edu.pk (I.A.)
[2] Department of Chemical Engineering, University of Malaya, 50603 Kuala Lumpur, Malaysia; badrules@um.edu.my (B.M.J.); raab@um.edu.my (R.I.)
[3] Institute of Electronic Structure and Laser, Foundation for Research and Technology-Hellas, N. Plastira 100, Vasilika Vouton, GR-700 13 Heraklion, Crete, Greece; stylianakis@iesl.forth.gr (M.M.S.); gkenanak@iesl.forth.gr (G.K.)
* Correspondence: raab@um.edu.my

Citation: Ahmad, W.; Jabbar, B.; Ahmad, I.; Janb, B.M.; Stylianakisc, M.M.; Kenanakisc, G.; Ikramb, R. Highly Sensitive Humidity Sensors based on Polyethylene Oxide/CuO/Multi Walled Carbon Nanotubes Composite Nanofibers. *Materials* **2021**, *14*, 1037. https://doi.org/10.3390/ma14041037

Academic Editor: Antonio Di Bartolomeo

Received: 18 January 2021
Accepted: 16 February 2021
Published: 22 February 2021

Publisher's Note: MDPI stays neutral with regard to jurisdictional claims in published maps and institutional affiliations.

Copyright: © 2021 by the authors. Licensee MDPI, Basel, Switzerland. This article is an open access article distributed under the terms and conditions of the Creative Commons Attribution (CC BY) license (http://creativecommons.org/licenses/by/4.0/).

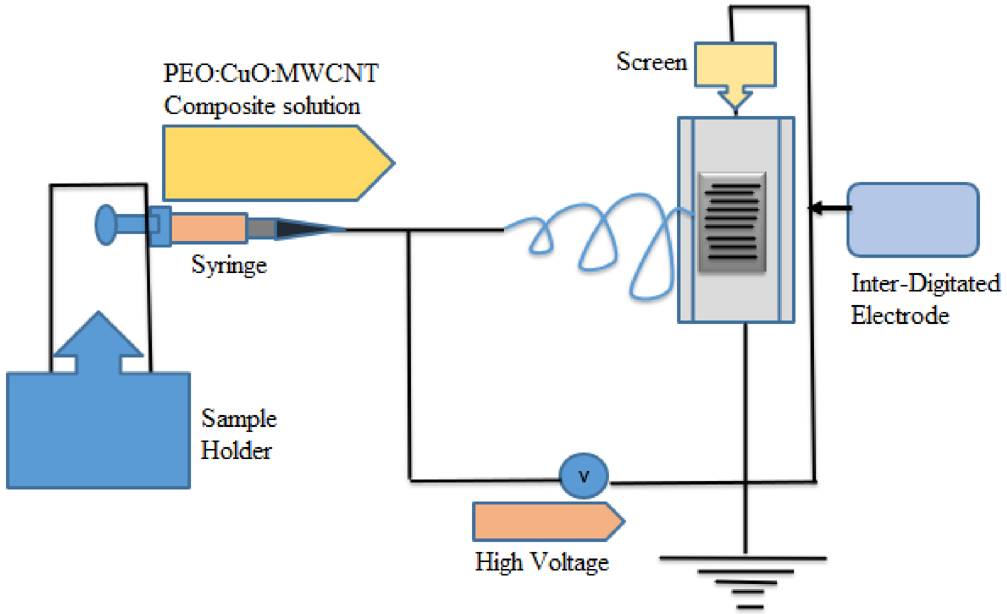

Figure S1. The schematic diagram of the Electrospinning process.

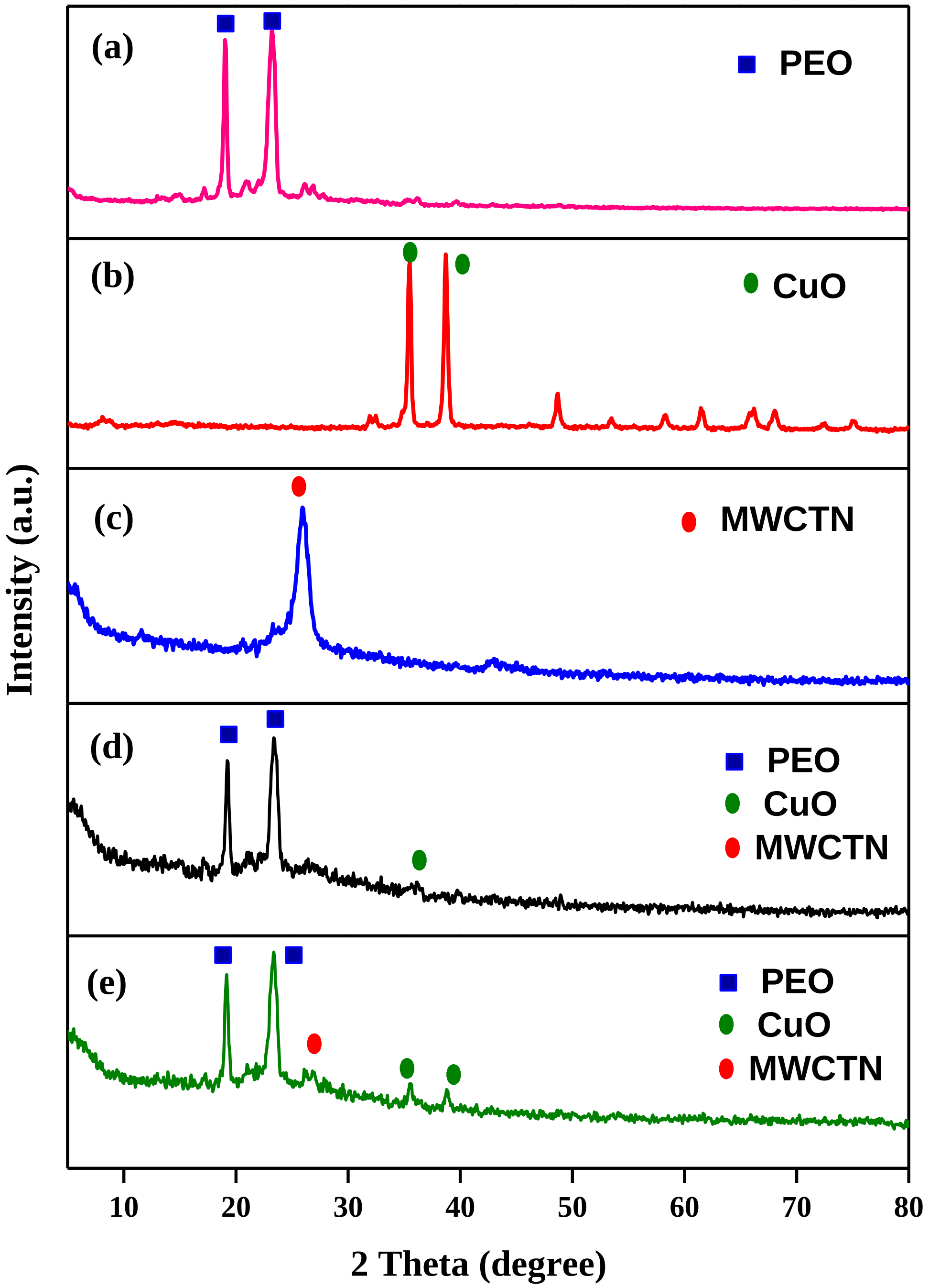

Figure S2. The XRD patterns of (**a**) pure PEO (**b**) CuO nanoparticles (**c**) MWCNT (**d**) PEO–CuO–MWCNT: 1% nanocomposite and (**e**) PEO–CuO–MWCNT: 3% nanocomposite.

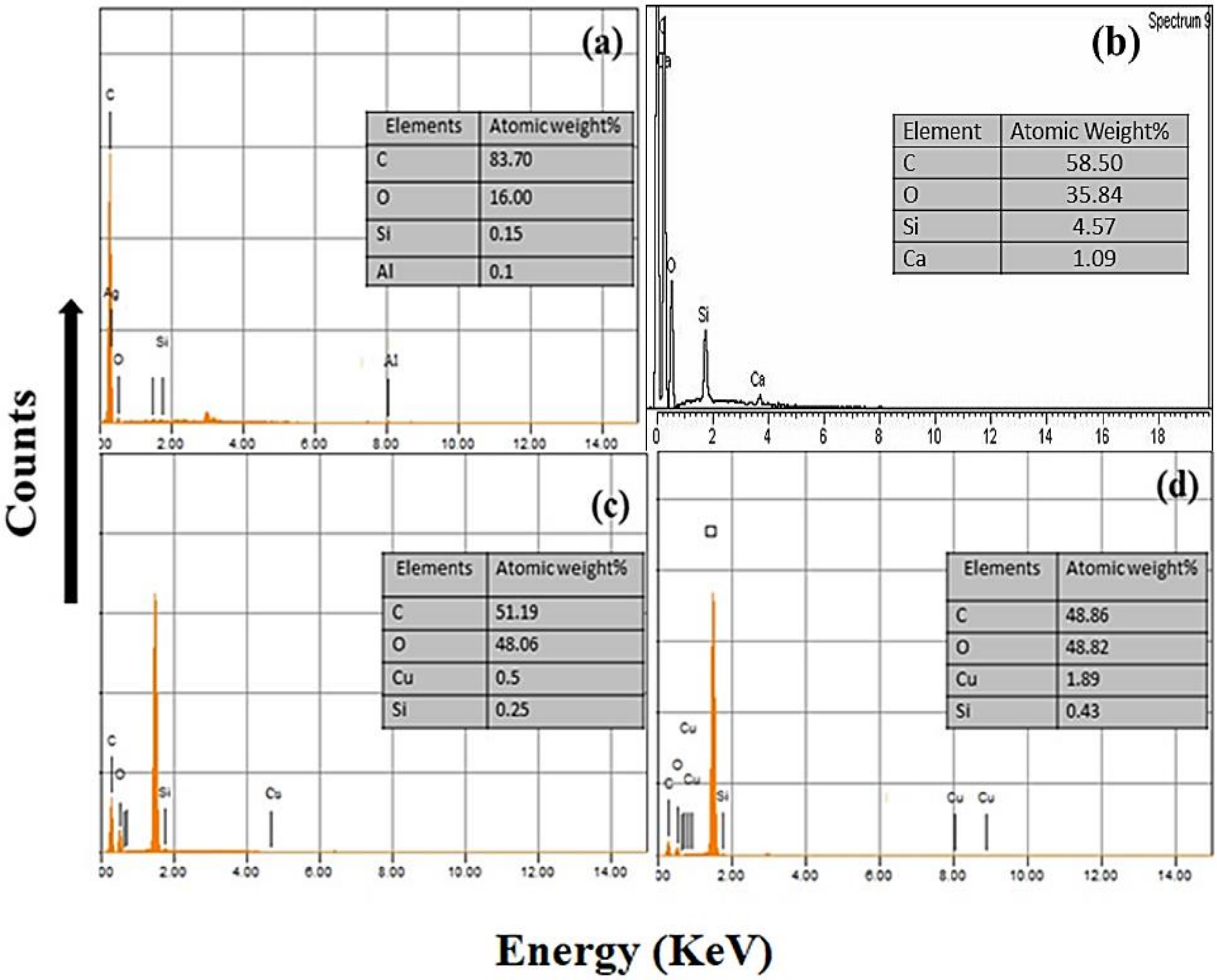

Figure S3. EDS elemental profiles of (**a**) MWCNT (**b**) pure PEO (**c**) PEO–MWCNT–CuO: 1% nanofibers, and (**d**) PEO–MWCNT–CuO: 3% composite nanofibers.
